# Neurotechnology: Bridging the dialogue between engineers, material scientists, clinicians, and ethicists

**DOI:** 10.1016/j.isci.2022.105432

**Published:** 2022-11-12

**Authors:** Flavia Vitale, Jennifer N. Gelinas, Laura Y. Cabrera

**Affiliations:** 1 University of Pennsylvania, Philadelphia, PA, USA; 2 Columbia University Irving Medical Center, New York, NY, USA; 3 Pennsylvania State University, University Park, PA, USA

## Abstract

This backstory is a conversation highlighting the importance of interdisciplinary collaboration for developing the field of neurotechnology and for its safe clinical translation and assessment of its societal impacts.

**Figure:**
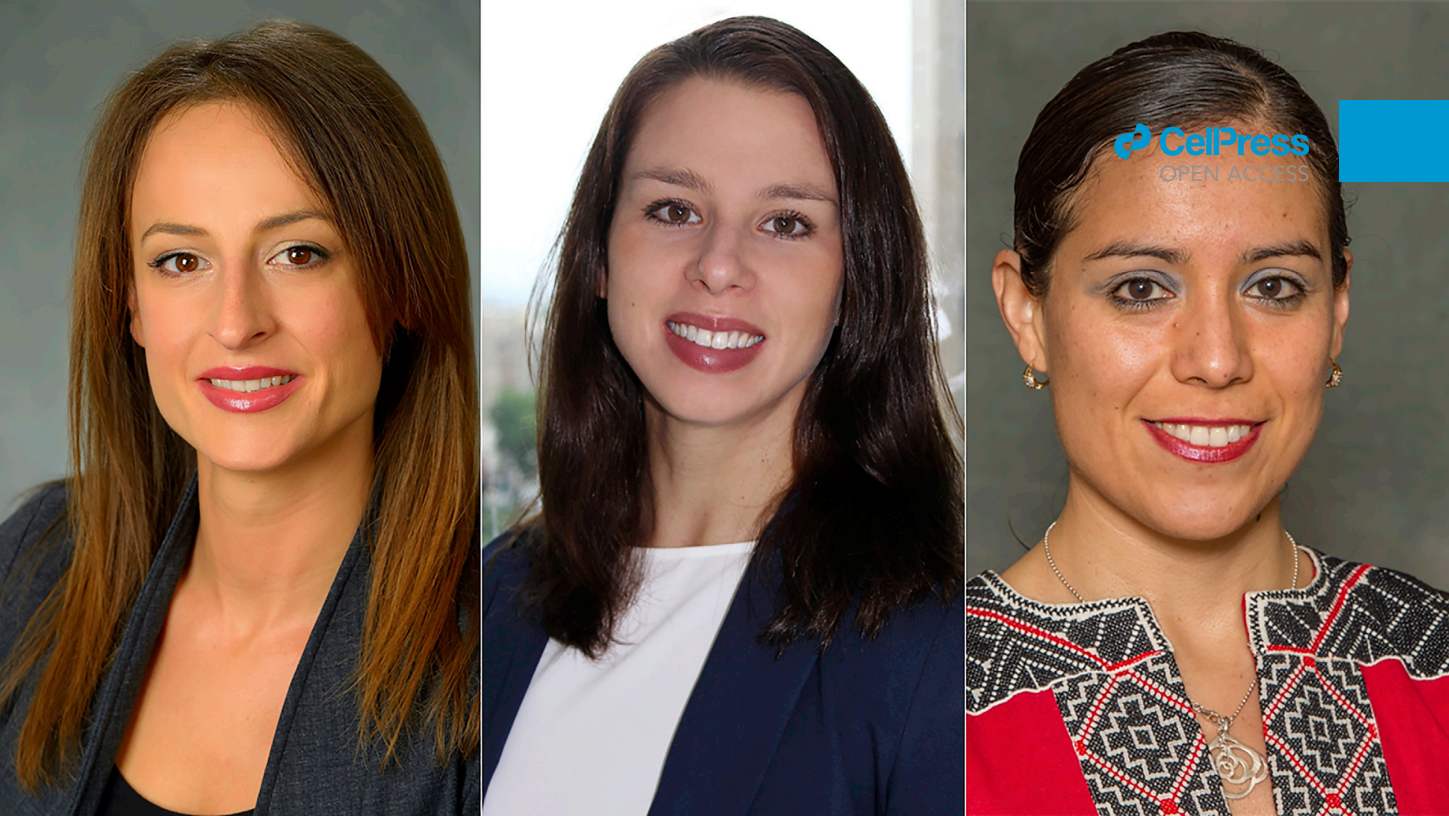
Leaders of the field (left to right): Dr. Flavia Vitale, Dr. Jennifer N. Gelinas, and Dr. Laura Y. Cabrera encourage interdisciplinary research and dialogue between different stakeholders to ensure the translation of these technologies to clinics and for its societal impacts.

It is difficult for any individual to acquire the experience and expertise to address all facets of the field. Therefore, it is critical for experts from different fields to work together to move the field forward.

When thinking of Neurotechnologies transitioning to clinics, it is essential to consider the known and unknown risks and side effects but also understand the attitudes and concerns of different stakeholders around them.

I would suggest being curious, rigorous, open-minded, and willing to learn the multiple technological and scientific concepts behind Neurotechnology design and development.

### Introduction

Neurotechnologies have experienced exponential growth in the past decade. Novel materials, molecules, techniques, and devices are constantly being developed to directly interface with the nervous system’s different components. Coupled with advances in understanding the mechanisms underlying neural functions and disease, these approaches can provide an important window into the nervous system and allow us to precisely manipulate its activity. Although such novel opportunities have the potential to enhance neural functions and treat neuropsychiatric diseases, we must be mindful that neurotechnologies interact with the nervous system—which is not fully understood in its complexity—and can have unexpected consequences, which need to be carefully evaluated also from an ethical and social perspective.

In this backstory part of the *iScience* Special Issue Neuronal engineering and implantable devices (https://www.sciencedirect.com/journal/iscience/special-issue/10J8BLPPRZ3), authors Dr. Flavia Vitale, Dr. Jennifer N. Gelinas, and Dr. Laura Y. Cabrera encourage dialogue between engineers, material scientists, clinicians, and ethicists. They also share their thoughts and personal experiences on the current challenges in the field, the importance of interdisciplinarity, and the societal impacts of Neurotechnology.

### Proximity

#### What is the motivation, and why do we need conversation between disciplines to advance research in neurotechnology and neuroengineering?

Flavia: Neuroengineering is inherently multidisciplinary, bringing together medical, neuroscience, engineering, materials, and data science expertise. Advancing neurotechnologies from bench to bedside requires large teams where this broad range of expertise is represented to ensure that every single aspect of the device is optimized for human translation.

Jennifer: It is difficult for any individual to acquire the experience and expertise to address all facets of the field. Therefore, it is critical for experts from different fields to work together to move the field forward. Otherwise, we risk moving forward in one aspect at the expense of another. Neurotechnologies interact with the nervous system—which is not fully understood in its complexity—and can have unexpected consequences that must be carefully evaluated.

Laura: The motivation is to develop the best science and engineering for social good. As we advance Neurotechnology, we must ensure that we have considered the scientific and engineering facts and how these advances will impact individuals and society. Thus, for Neurotechnology, a responsible manner needs interdisciplinary conversations among engineers, material scientists, clinicians, and ethicists (and other disciplines).

How do individual scientists or groups fit into the interdisciplinary nature of the Neurotechnology and Neuroethics field? How can one bring together such broad disciplines?

Jennifer: Individuals and/or individual research groups are the building blocks for advancement in the field. Open communication and the attitude surrounding collaborations between fields are key. Rather than working individually with proprietary devices, protocols and methods should be shared so that groups can effectively build upon the work of others and quicken the pace of progress.

Laura: Besides collaboration between clinicians and applied natural scientists, we also need social scientists (like anthropology and sociology) and lawyers joining the trenches. Often each discipline has its way of operating, and it is important to acknowledge those differences and find ways to move together. Increasingly people are becoming interdisciplinary by training. I, for example, trained as an electrical and communication engineer and then transitioned to applied ethics, settling finally into Neuroethics, where I have focused my interests on the intersection of Neurotechnology, the brain, and the ethical and social implications of Neurotechnology.

### Language

#### What are the challenges in communication between disciplines, and how can we solve them?

Jennifer: Disciplines are typically built on a large amount of foundational knowledge that becomes so well-known and well-used to the individuals who practice within this discipline that they begin to lose the notion that anyone could not be aware of this knowledge. These differences in foundational knowledge can lead to incorrect assumptions and a lack of expectation matching within collaborative projects. Seminars with a didactic component aimed at teaching the basics of a field to a broad audience can be invaluable in allowing sharing of key foundational knowledge. Shared laboratory meetings between groups within different disciplines can expose researchers and their trainees to the practicalities of research in a field distinct from their own.

Laura: Collaborating in interdisciplinary teams brings several challenges in communication. For example, there are terms that each discipline uses with different meanings. I will give you two examples. In one of my grants, I have focused on exploring various stakeholders' attitudes and views toward psychiatric electroceutical interventions. In one of our manuscript submissions, one of the reviewers asked us to drop the use of the term “electroceutical,” as, according to the reviewer, that was a made-up word. Yet we did not come up with the word, someone else did, and we thought it was a suitable term to contrast with pharmaceutical, where “pharma” points to the chemical nature of the intervention so that “electro” would reflect the electrical nature of the intervention. Another example from that project is that one of our scales measures affect. For the sociologist, the philosopher, and myself, using the term affect to describe a scale measuring how a given intervention makes participants feel makes perfect sense. Yet, our psychiatrist colleague reminded us that in psychiatry, “affect” is used very differently, so we had to be sure we described in our methods when publishing for that audience how we were using the term affect.

### Research methods

#### Describe your approach to develop or adjust the methodology for advancing neurotechnology leading to clinics

Flavia: We closely interact with clinicians and surgeons who help us identify the unmet clinical needs, users, and operational workflows. Based on these requirements, we then define the technical specifications from the engineering and design standpoint. In every step of the device development, testing, and validation, we ensure that the devices meet the specifications from the technical, functional, safety, and usability standpoints based on our clinical collaborators' feedback.

Laura: In my field of Neuroethics, the approach I have taken is to understand different stakeholders involved in developing a given Neurotechnology for clinical use. For example, we have done interviews with a variety of providers, patients, and caregivers. Even involving the public is relevant as their taxpayer money is the one likely funding some of these developments.

#### How do you prepare your students/early career researchers in this field?

Flavia: My trainees come from various backgrounds, and they are fully in charge of every aspect of their research project, ranging from device design, fabrication, and testing to the execution of the *in vivo* studies. This ensures that they can get trained in different aspects of neuroengineering research, while also getting rigorous training in biomedical science methods.

Jennifer: We prepare our mentees for interdisciplinary research by exposing them early and consistently. We have a joint laboratory meeting between a primary engineering and neurology research group that encourages them to ask critical questions. We often pair trainees from different backgrounds to work together on collaborative projects requiring them to pool their skills to move forward. They present at interdisciplinary conferences in addition to their specialty conferences. We review and provide feedback on their presentations, emphasizing how to make their project rationale and results accessible to a broad audience.

Laura: Most of my undergraduate students come from neuroscience or human biology majors without much experience in qualitative methods and in some cases, no prior exposure to ethics. So, we have them read publications on ethics on qualitative methods and provide them with hands-on experiences to develop new skills (e.g., carrying qualitative content analysis, identifying key ethical concerns, and developing their arguments logically). Most of the people joining my laboratory are curious and not afraid to get out of their comfort zone; they enjoy learning new things and complementing their skill sets, and we nurture that desire while guiding them in their learning path.

### Publication and governance

#### What to consider for publishing papers in Neurotechnology and Neuroethics?

Flavia: Because of the nature of the work, the primary outlets for Neurotechnology publications are typically interdisciplinary journals targeting a broad audience. This can make the selection of the target journal a bit challenging. In recent years, however, there has been a significant increase in publications in more technical engineering journals, primarily those with a materials science and nanotechnology focus.

Laura: One must be clear on the audience one aims to reach. In my laboratory, we try to place some publications in clinical or technology-oriented journals and some in more social science/ethics journals. That makes a big difference in how one writes a paper and frames the key findings. Deciding where a given paper might have the biggest impact is not always clear, but it is definitely an important consideration. Finally, suggesting reviewers who are open to an interdisciplinary lens or who can cover the different areas that a paper entails can also be challenging.

#### How do you think the governance of interdisciplinary projects (e.g., getting funding, project planning, and management) impacts the research in Neurotechnology and Neuroethics?

Jennifer: Governance strongly affects how Neurotechnology research is conducted. Especially for human subject research, the cost of bringing novel devices through the appropriate approval process can be completely prohibitive without specific funding sources. Similarly, the number and diversity of personnel required to run these projects effectively can favor large institutions with well-established infrastructure. These requirements can make it difficult for smaller groups to “break in” to the field. Similarly, institutions without strong connections between engineering, science, and medical departments can have problems coordinating the distribution of funds, personnel training, and data sharing.

### Societal impacts of neurotechnology

#### What are the challenges for neurotechnology from the perspective of personalized medicine?

Jennifer: Personalized medicine requires an in-depth understanding of what individual features are relevant to the treatment in question and which could be extraneous. There will always be limits to how much a drug or device can be customized for an individual, whether that is based on science or economic feasibility. It will be essential to strike a balance between the most effective treatments for patients with the least side effects. For instance, an expensive genetic therapy that is effective for one particular variant in a gene that is expressed by just a few patients worldwide would face severe feasibility challenges compared with a therapy that is effective for a multitude of variants in the same gene, expressed by many more patients. What if the latter treatment carried a slightly higher risk of side effects? Questions such as “how would the risk/benefit profile be established for this group of patients, and who would decide which therapy moves forward for commercialization” become critical.

Laura: How neuromodulation and neurostimulation interventions are used or intended for psychiatric disorders brings challenges related to the stigma around psychiatric disorders and around some types of interventions, as is the case for electroconvulsive therapy. Although deep brain stimulation is used widely for patients with movement disorders, there is still hesitation and concern from the public regarding its use of psychiatric disorders. In the case of adaptive implants, some people like the idea of an implant that can respond to their brain signals and adjust the electrical stimuli accordingly; others dislike the idea of having an implant making those adjustments as they do not trust an implant the same way they do their clinician.

#### What are the considerations and challenges to improve uptake of Neurotechnology for rehabilitation?

Jennifer and Flavia: One major challenge is ensuring equity in access to rehabilitation. Many such therapies are only available through certain research institutions or specialized clinics. Socioeconomic factors may therefore play a role in who has access to care. Next, invasive approaches to rehabilitation are typically tested in only the most severely affected individuals due to the potential risks (for instance, implanting a neural interface device in a completely paralyzed patient rather than a patient with partial paralysis—what if the device worsened the paralysis?). The device may be easier to implement in a less severely affected individual, and this individual may desire access to the therapy. However, the primary consideration in the medical field is first to do no harm. How we weigh the pros and cons of invasive rehabilitation therapies will differ depending on the perspective. Finally, it is critical to “bring on board” therapists and other clinical support staff, who play a central role in the rehabilitation process. If we can show them the advantages of adopting Neurotechnologies in the rehabilitation practice, it will significantly lower barriers to adoption, dissemination, and access.

Laura: When thinking of Neurotechnologies transitioning to clinics, it is essential to consider the known and unknown risks and side effects and also understand the attitudes and concerns of different stakeholders around them. Are there misconceptions that need to be addressed before they can transition to the clinic, ensuring uptake? Are concerns similar among non-clinical groups and clinical groups? If not, what might be the reason for the dissonance, and how to best address it? Are we considering end-user values and preferences in the design and use of the technology? If this Neurotechnology was widely used, what types of values does it promote in society? Does it bring power imbalances that need to be checked? Does it create or exacerbate inequalities?

#### What are the educational approaches to increase public awareness?

Flavia: The general public is becoming increasingly aware of Neurotechnologies, thanks to the increase in the number of companies investing in developing and commercializing these products. However, the communication around these tools often tends to emphasize “Sci-Fi” aspects rather than their potential benefits for patient care, quality of life, and outcomes. This significantly affects the public perception of Neurotechnologies, as it can raise skepticism and ethical concerns.

Jennifer: Public awareness of the potential for personalized medicine and neurorehabilitation can be increased by the participation of public figures in the field. Although beneficial in generating interest (and potentially funding) for ongoing research/clinical endeavors, this approach does not always lead to a practical understanding of what such technologies can offer to an individual. Many institutions now have easy-to-use clinical trials websites that provide information regarding ongoing trials and recruitment information. This approach allows patients to directly interface with individuals conducting cutting-edge research about disorders that affect them.

Laura: Better educational materials that integrate the views and concerns of non-clinical groups, like animations or other interactive approaches (perhaps virtual reality or augmented reality approaches or apps). Make sure that the educational strategies have a balanced portrayal—not too controversial and not so optimistic that they get hyped. Finding this balance could be considered a challenge.

### Future

#### What did you learn about interdisciplinary research on Neurotechnology and Neuroethics, and what tips would you give anyone considering undertaking such projects?

Flavia: I would suggest being curious, rigorous, open-minded, and willing to learn the multiple technological and scientific concepts behind Neurotechnology design and development. I would also encourage trainees to get exposed to clinicians and patients to understand better the pipeline, processes, and potential barriers around the adoption and integration of Neurotechnologies in the clinical workflows. Such connections will also help identify and understand unmet needs that new technologies can address.

Laura: I have learned so much from my colleagues; it is incredible to see how their perspective improves our science. In my case, putting my team together, having a psychiatrist with expertise in at least one of the PEIs that we were studying; a sociologist with expertise in conducting national surveys, with interest in emerging technologies; and a philosopher interested in Neuroscience and Neurotechnology was vital. From an ethical point of view, having social scientists and humanists collaborating with scientists and engineers helps ensure we align scientific progress and technological innovation with societal values and priorities.

